# Universal conformational properties of polymers in ionic nanogels

**DOI:** 10.1038/srep19836

**Published:** 2016-02-01

**Authors:** Hideki Kobayashi, Roland G. Winkler

**Affiliations:** 1 Theoretical Soft Matter and Biophysics, Institute for Advanced Simulation, Forschungszentrum Jülich, 52425 Jülich, Germany

## Abstract

Polyelectrolyte gels are known to undergo significant conformational changes in response to external stimuli such as pH, temperature, or the dielectric constant. Specifically, an increase of the degree of ionization associated with an increasing number of counterions leads to swelling of the network. For a macroscopically large gel, which is electrostatically neutral in its interior, swelling is no longer governed by electrostatic interactions, but rather by the osmotic pressure of counterions. However, this electrostatic neutrality is typically violated for nanogels, because counterions are free to leave a gel particle. Although nanogel-swelling exhibits similar features as swelling of micro- and macrogels, another mechanism has to be relevant. Here, we use molecular dynamics simulations and scaling theory to unravel the structural properties of nanogels upon changing the electrostatic interactions. We demonstrate that the swelling of nanogels is governed by screened electrostatic interactions without a relevant contribution by the counterion osmotic pressure.

Nano- and microgels are nanometer to micrometer size crosslinked polymer networks often comprised of polyelectrolytes. Their ability to undergo reversible volume phase-transitions in response to environmental stimuli, such as pH, temperature, the ionic strength of the surrounding medium, or the quality of solvent^1^,^2^ renders them potential candidates for a broad-range of applications in drug delivery, sensing, template-based synthesis of inorganic nanoparticles, and separation and purification technologies^3^,^4^,^5^,^6^,^7^,^8^,^9^ to name a few. Numerous theoretical and simulation studies of the swelling behavior of polyelectrolyte networks have been performed, in order to arrive at a microscopic understanding of the underlying mechanisms^10^,^11^,^12^,^13^,^14^,^15^,^16^,^17^. These studies typically focus on defect-free macrogels applying periodic boundary conditions, i.e., only the bulk properties of a gel are considered^18^.

Comparably little is known about finite-size crosslinked polyelectrolyte nano- and microgels^19^,^20^,^21^,^22^,^23^. Their finite size gives rise to phenomena, which are not present in bulk systems. Specifically, the permeability of a gel particle allows counterions to freely penetrate or leave the gel particle in response to the actual charge distribution and environmental conditions. For weak electrostatic interactions, no longer all counterions are contained inside the gel particles, as for a bulk system, but rather a large fraction is distributed in its vicinity^19^,^21^,^24^. In contrast, strong Coulomb attractions lead to counterion condensation and capturing of counterions inside a gel particle, comparable to macroscopic gels. The change of the counterion density associated with the repulsive Coulomb interaction between monomers leads to an interesting interplay, which significantly affects the nano- and microgel structural properties.

The specificities by the finite size are reflected in the over-all charge of a gel particle. Macroscopic gels can be considered as neutral, at least their major part aside from a thin surface layer. This is in contrast to nano-and microgels. Below a certain size, the nanogels are no longer neutral, there is not even a neutral “core” part^19^,^21^. It is this type of gels, which we are interested in and will denote as nanogels in the following. The non-neutrality has far reaching consequences for gel swelling. In a bulk gel, which can be considered as electrostatically neutral, there is a broad range of electrostatic interactions, where gel swelling is attributed to the osmotic pressure of the counterions^25^,^26^,^27^. Such a mechanism does not apply for nanogels, since counterions are able to leave the gel particle. Here, the question arises on the mechanisms which govern gel swelling in such systems, particularly on the range of interactions, where macrogels swell by counterion osmotic pressure.

Other finite-size branched polyions of different topology, e.g., stars, micelles, and brushes, share certain similarities with nanogels^24^,^28^. Specifically the permeability is present. However, there are also distinct geometrical differences, such as the charge distribution of star polymers, which increases toward the star center and gives rise to particular conformational properties^28^.

In order to elucidate the structural properties of nanogel particles, we perform large-scale computer simulation, combining molecular dynamics simulations for the polymers with the Brownian multiparticle collision dynamics (B-MPC) approach^29^,^30^. Counterions are taken into account explicitly. Our emphasis is on the conformational properties of the polyelectrolyte chains. We identify various swelling regimes. For weak electrostatic interactions, the so-called *unscreened* regime is obtained^28^, where the polymers swell with increasing Coulomb interaction strength. Here, we find that swelling is independent of the presence or absence of counterions. We derive a novel scaling relation, which captures the dependence of swelling on the Bjerrum length, number of crosslinks, and polymers. This regime is followed by a regime, which we denote as *screened*, in contrast to the typical notion of *osmotic* regime^26^,^28^. We show that screened electrostatic interactions are responsible for the observed conformational polymer properties. For even larger interaction strengths, counterions condense at the polymers and the nanogel collapses^31^.

## Results

### Computer simulations

We consider a model nanogel comprised of a regular network of *N*_*p*_ polyelectrolytes connected by *N*_*c*_ tetra-functional crosslinks. An individual polyelectrolyte is described as a self-avoiding linear chain composed of *N*_*m*_ coarse-grained monomers^29^. Every monomer carries a charge *q*_*m*_ = *e*. Counterions of charge *q*_*c*_ = −*e* are taken into account explicitly, such that the whole system is electrostatically neutral. The electrostatic interactions are described by the Coulomb potential. We characterize the strength of the Coulomb interactions by the interaction parameter Γ = *e*^2^/*εlk*_*B*_*T* = *l*_*B*_/*l*, where *ε* is the dielectric constant of the implicit solvent, *k*_*B*_ the Boltzmann constant, *T* the temperature, *l*_*B*_ = *e*^2^/*εk*_*B*_*T* the Bjerrum length, and *e* the magnitude of the elementary charge. Variations of Γ correspond to changes of the charge density via their separation *l* or the Bjerrum length. We consider nanogels with *N*_*p*_ = 220 and *N*_*p*_ = 1236 polymers, each with *N*_*m*_ = 20 monomers, with the corresponding numbers of crosslinks *N*_*c*_ = 147 and *N*_*c*_ = 729.

**Figure 1:**
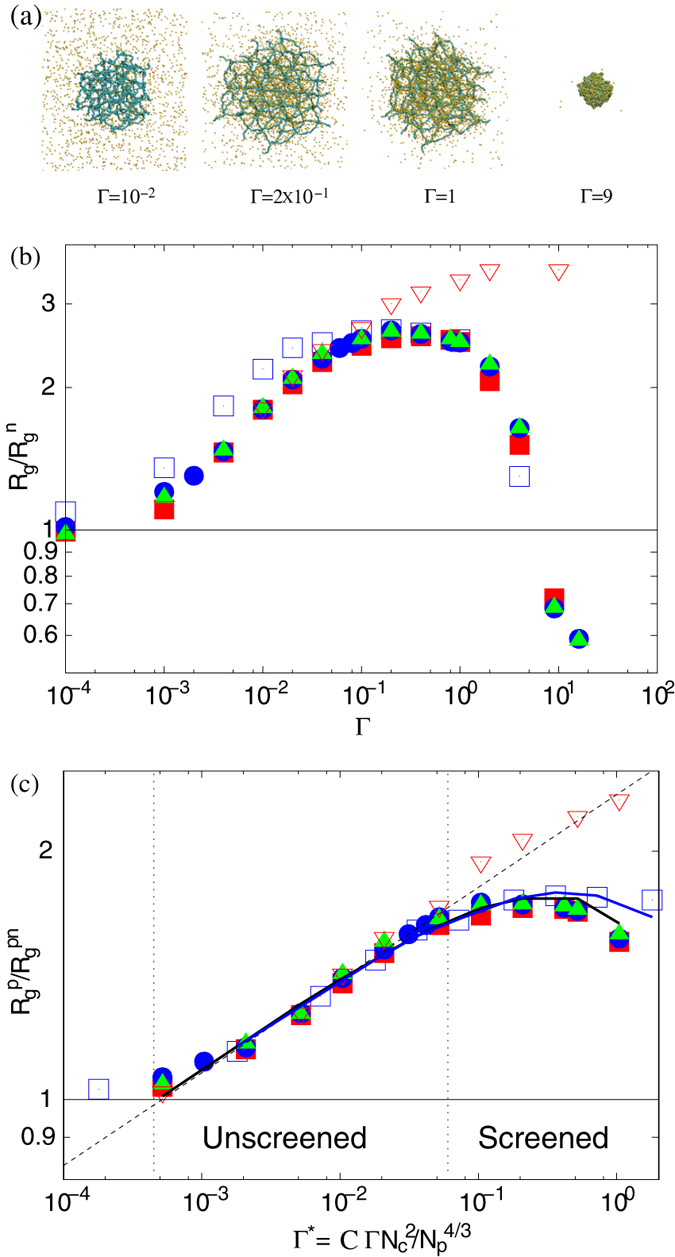
(**a**) Snapshots of gel particles in the presence of counterions (yellow) for various Coulomb interactions strengths Γ as indicated. (**b**) Dependence of the nanogel radius of gyration *R*_*g*_ on the interaction strength Γ for *N*_*c*_ = 147 and the system sizes *L*/*l* = 200 (■), 300 (•), 400 (▲), and *N*_*c*_ = 729 for the systems size *L*/*l* = 400 (⌌). The symbols ∇ indicated simulation results without counterions for *N*_*c*_ = 147. 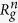 is the radius of gyration of a neutral gel, where 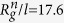 for *N*_*c*_ = 147 and 31.0 for *N*_*c*_ = 729. (**c**) Average radii of gyration of individual polymers 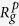 as function of the effective interaction strength Γ^*^. The dashed line is proportional to (Γ^*^)^0.12^. The solid lines are the numerical solutions of the equation ∂*F*/∂*R*_*E*_ = 0, with *F* of Eq. (12). *C* = 0.032 for *N*_*c*_ = 147 (black) and 0.045 for *N*_*c*_ = 729 (red). 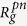 (= 2.6 *l*) denotes the average radius of gyration of individual polymers.

**Figure 2:**
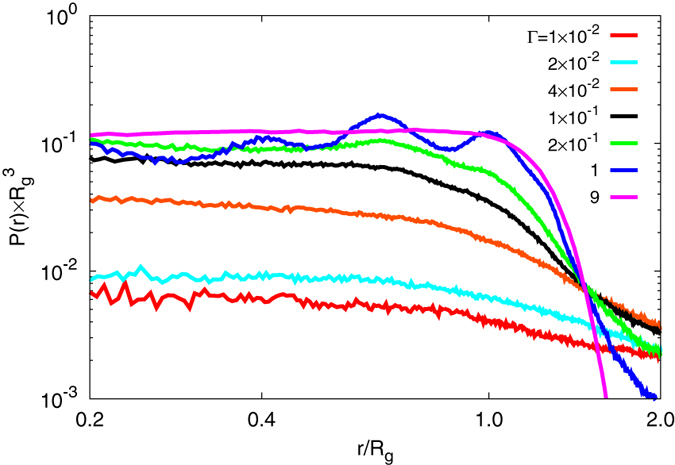
Normalized radial counterion distribution functions *P*(*r*), with respect to the nanogel center of mass, for various interaction strengths as well as *N*_*c*_ = 147, *N*_*m*_ = 20, and *L*/*l* = 300. *R*_*g*_ is the nanogel radius of gyration for the respect value of Γ (cf. Fig. 1(b)).

Figure 1(a) shows snapshots of gel particles for various interactions strengths. For 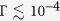, polymers behave as in a neutral system and the counterions are rather homogeneously distributed over the whole available volume (see also Fig. 2). With increasing Γ, the ion density inside the nanogel increases, and for Γ > 1 the counterions start to condense along the polymers. Thereby, the nanogel radius of gyration *R*_*g*_ and that of the individual polymers 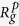 increases first, passes trough a flat maximum and decreases again for Γ > 1, as shown in Fig. 1(b). It is exactly the appearance of this plateau-like regime, which needs to be understood microscopically. Eventually, the nanogel collapses to a size smaller than that of a neutral nanogel due to counterion-mediated attractions of equally charged monomers^20^,^31^. A qualitatively similar behavior has been obtained for nanogels in ref. 20.

We consider dilute solutions of nanogels. In the plateau-like regime of interaction strengths, the gel radius of gyration is 
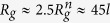
. This corresponds to the packing fractions 5 × 10^−2^ and 6 × 10^−3^ for the system sizes *L*/*l* = 200 and 400, respectively. In this range of system sizes, the gel size is rather independent of the nanogel concentration. The increase of the simulation box size by a factor of two, i.e, a change in concentration by a factor eight, hardly changes the shape of the radius of gyration curves. Since the counterion concentration is rather low outside of a nanogel in the plateau-like regime (cf. Fig. 2), we expect very little concentration effect on the gel conformation in this interaction range as long as the nanogel concentration is smaller than the overlap concentration. Interestingly, up to Γ ≈ 0.1, the size of the gel particles is independent of the presence or absence of counterions, since *R*_*g*_ increases essentially in an identical manner in systems without counterions. Hence, counterions neither contribute to gel swelling nor shrinkage in this regime. This is in strong contrast to bulk gels, which exhibit a significant influence of the counterions on gel swelling^13^,^15^ and is related to the nanometer size of the permeable particle. Here, a crossover from our particle-type behavior to bulk-gel behavior will appear above a certain particle size.

We find a pronounced dependence of 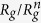 on *N*_*c*_ and, hence, *N*_*p*_. Nanogels with *N*_*c*_ = 729 are more swollen at small Γ, but the nanogel-size saturates at about the same relative radius of gyration. We attribute the stronger polymer stretching at small Γ to the larger amount of charges of the larger nanogel. We will address this aspect again further below.

Figure 2 shows radial counterion distribution functions *P*(*r*) for various interactions strengths Γ. The density of counterions within a gel particle (*r*/*R*_*g*_ < 1.5) increases with increasing Γ and decreases at the same time in the outer volume. For large Γ, the electrostatic interactions between charged monomers and counterions effectively trap a major fraction of them inside of the nanogel. In contrast, for Γ < 2 × 10^−2^ the inside an outside counterion densities are rather similar. This implies that the osmotic pressure due to counterion-density differences is rather small or even negligible and cannot affect the swelling behavior of nanogels, at least in this regime.

**Figure 3:**
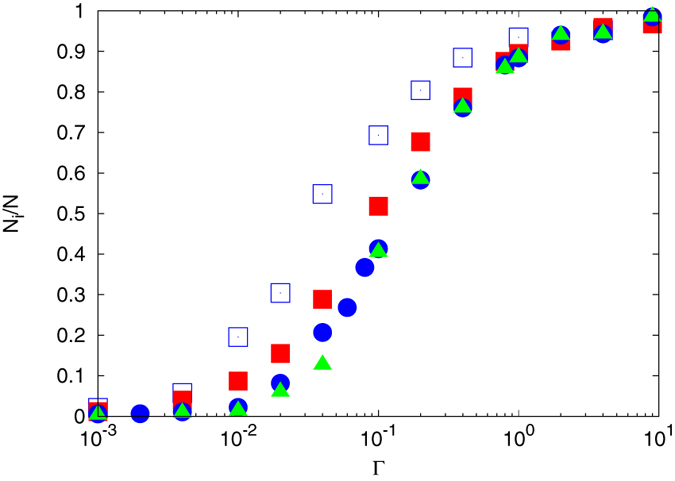
Fraction of counterions inside (*r* < 1.5*R*_*g*_) a nanogel as a function of the interaction strength. The parameters and symbols are the same as in Fig. 1. *N*_*i*_ is the number of counterions within a gel particle, and *N* = *N*_*m*_*N*_*p*_ + *N*_*c*_. The total number of counterions is *N* = 4547 for *N*_*c*_ = 147 and 25549 for *N*_*c*_ = 729. Note that the number of outside ions is 1 − *N*_*i*_/*N*.

**Figure 4:**
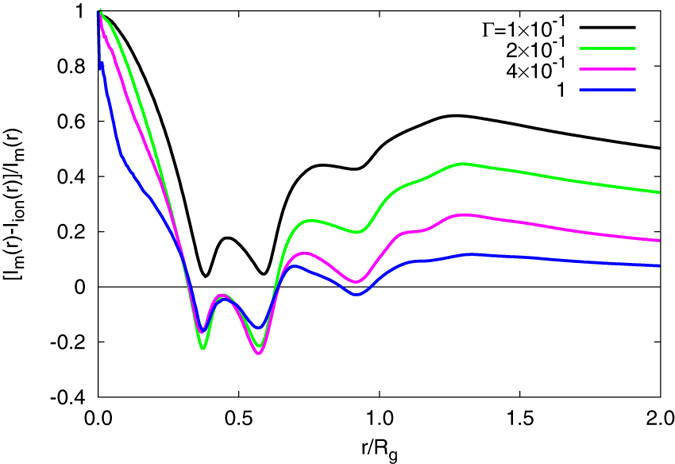
Charge-difference ratios (cf. Eq. (1)) between monomers and counterions as function of their radial distance from the nanogel center for the indicated interaction strengths. The systems size is *L*/*l* = 300.

Figure 3 displays the fraction *N*_*i*_/*N* of counterions inside of a nanogel, i.e., within the radial distances *r* < 1.5*R*_*g*_ with respect to the center-of-mass of the particle^32^. Here, *N*_*i*_ is the number of counterions within a gel particle and *N* = *N*_*m*_*N*_*p*_ + *N*_*c*_ is the total number of charged monomers. The homogeneous ion distributed for Γ < 10^−3^ (cf. Fig. 2) implies a rather small ratio *N*_*i*_/*N*. With increasing Γ, the interior concentration increases monotonically and approaches unity for Γ > 10 due to Manning condensation^33^. The counterion concentration of the larger particle with *N*_*c*_ = 729 exceeds that of the smaller one for almost all Γ. Again, this is attributed to stronger Coulomb interactions by the higher total charge of the larger particle. In contrast to the gel radius of gyration *R*_*g*_ and the polymer radius of 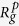 displayed in Fig. 1(b,c), *N*_*i*_/*N* naturally strongly depends on the simulation-box size, because a larger volume corresponds to an overall smaller counterion concentration. However, for 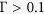, the concentration effect disappears and the Coulomb interactions begin to dominate over the counterion translational entropy. Electrostatic interactions between the monomers and the counterions effectively trap a fraction of them inside the nanogel. However, their density is lower than that of monomers for 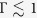. This has already been reported in ref. 19, where the dependence of the number of counterions inside a nanogel has been studied as function of gel size. Figure 4 illustrates in more detail the radial effective charge. Here, ratios of the difference between the radially integrated monomer and counterion charges and the respective integrated monomer charge density are shown. The radial charge is calculated according to


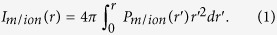


Evidently, the ratio is always positive for Γ = 0.1. Hence, the nanogel interior is oppositely charged compared to the counterions over the whole volume. To be precise, this applies for all interaction strengths 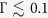. For 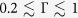, there is a certain volume inside of the gel in the vicinity of *r*/*R*_*g*_ ≈ 0.5, where the counterion charge dominates over the monomer charge. Note that the actual shape of the effective charge depends on the network structure. Since the gel extends up to *r*/*R*_*g*_ ≈ 1.5 (cf. Fig. (2)), the volume of the positively charged gel fraction exceeds the screened volume by far. Thus, the net-charged nanogel volume comprises a large volume fraction of the gel particle. Again, this is in stark contrast to models, which explain swelling of randomly branched polyelectrolytes by the counterion osmotic pressure and assume electrostatic neutrality^26^.

### Scaling theory

**Figure 5:**
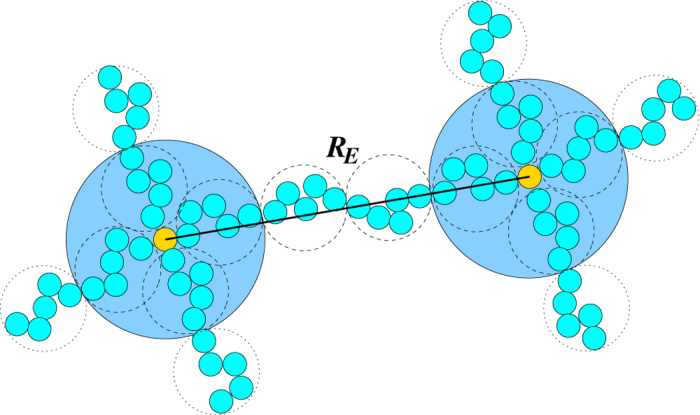
Schematic illustration of the tension blob model of crosslinked polymers. The Coulomb repulsive interactions between the four blobs at crosslinks leads to polymer stretching. The solid line indicates the mean distance *R*_*E*_ between crosslinks.

To arrive at a quantitative understanding of the swelling behavior of nanogels, we perform a scaling analysis of the average deformation of individual polymers. Average radii of gyration of individual polymers are displayed in Fig. 1(c). We will address two regimes, the *unscreened* regime and the *screened* regime. We adopt the tension blob model^34^ to characterize the polymer stretching, where the conformational properties of an individual polymer can be described by a sequence of *N*_*m*_/*g* blobs containing *g* monomers (cf. Fig. 5). Then, the average polymer extension is


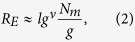


with *ν* ≈ 0.6 for a good solvent. If the polymer stretching is dominated by the electrostatic interactions between tension blobs, the stretching force of an individual polyelectrolyte chain is


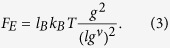


The size *lg*^*ν*^ of a tension blob is determined by the competition between its thermal energy and stretching by an external force *F*_*e*_. More precisely,


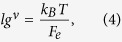


which is the length scale, where the free energy due to stretching is approximately equal to *k*_*B*_*T*^34^. By setting *F*_*e*_ = *F*_*E*_ and with Eq. (2), we find the scaling relation *R*_*E*_ ∝ Γ^(1−*ν*)/(2−*ν*)^. With the exponent *ν* = 1/2 of a theta solvent, the exponent becomes 1/3 as is well known by previous works^34^,^35^. This is just the scaling considerations of a single isolated polyelectrolyte. The specific properties of gel particles have to be taken into account to derive appropriate scaling relations of the conformational properties of their polyelectrolytes.

Due to the network structure, there is a higher polymer and charge concentration at the crosslinks, as illustrated in Fig. 5. We assume that the stretching of a polymer is dominated by the electrostatic forces between the respective four blobs around the crosslinks at their ends. These interactions will dominate over the forces by the other blobs along a polymer. Our model evokes reminiscences of the pearl-necklace structure of polyampholytes^36^ or polyelectrolytes in a poor solvent^37^,^38^. However, for our system, the physical origin is very different and arises from the geometry of the network structure. It is not self-organized as for pearl-necklace structures, but rather enforced by the geometry.

The electrostatic interactions between the blobs yield the stretching force


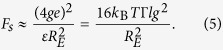


Equating this force with the force of the tension blob *F*_*e*_ of Eq. (4) and eliminating *g* via Eq. (2), we find the scaling relation for the average stretching of polymers


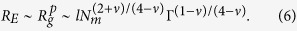


As shown in Fig. 1(c), the relation describes the simulation data extremely well. Our exponent (1 − *ν*)/(4 − *ν*) is significantly smaller than that derived above for an isolated polyelectrolyte chain. This underlines that a polymer of a nanogel behaves differently from a free polyelectrolyte, i.e., crosslinks play an important role in nanogel swelling.

We can generalize the result by approximating the electrostatic energy for polymer stretching by consider a gel particle as a conglomerate of spherically packed crosslinks of four blobs. This yields the total stretching energy


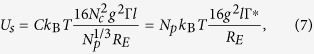


where we introduced the effective interaction strength


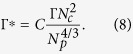


The factor *C* is determined by the structure of the nanogel. With the conformational free energy^34^


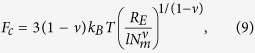


of an individual polymer and the electrostatic energy of Eq. (7), the polyelectrolyte free energy is


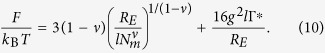


Calculation of the extremum, i.e., setting ∂*F*/∂*R*_*E*_ = 0, yields with Eq. (2)


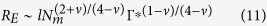


as generalization of Eq. (6). With the effective interaction strength, we arrive at a scaling representation of the polymer stretching for systems with different polymer and crosslink numbers. As shown in Fig. 1(c), our simulation results can well be scaled by the relation (11) in the *unscreened* regime.

As pointed out before, in the *screened* regime, the nanogel is not neutral and we suggest that gel swelling is caused by the repulsive Coulomb interactions between monomers. However, the present counterions screen the electrostatic interactions to some extent. To describe the polymer conformations in that regime, we replace the Coulomb energy in Eq. (10) by the Debye-Hückel energy, i.e, the free energy becomes


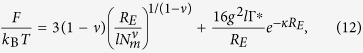


with the inverse Debye length 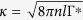 comprising the average counterion density *n* inside the nanogel. We assume that *n* is essentially independent of *R*_*E*_, which is confirmed by simulations.

Again, *R*_*E*_ is determined by the condition ∂*F*/∂*R*_*E*_ = 0. In the limit *κ* → 0, we recover the relation (11). The full numerical solution for the two considered networks of different size are presented in Fig. 1(c). Evidently, the simulation data are very well described by the screened Coulomb interaction in both, the *unscreened* and *screened* regime. We like to point out that there is only one fit parameter, namely *C* in our theoretical description. All other input quantities, such as the counterion density are taken from simulations. The latter produces the small differences between the theoretical curves in the vicinity of their maxima. Interestingly, Eq. (12) predicts the same maximum extension of a polymer independent of the gel size, i.e., *N*_*c*_ and *N*_*p*_, in agreement with our simulations.

We conclude that the conformations of polyelectrolytes in a nanogel in the *screened* regime are clearly determined by Coulomb interactions and charge screening. From our point of view, the quantitative agreement between theory and simulations rules out the hypothesis that nanogel swelling in the *screened* regime is caused by the osmotic pressure of counterions.

## Discussion

In this paper, we adopted a coarse-grained description of a polymer, where, as often in polymer physics^39^, a segment of length *l* comprises many “real” monomers, and characterized the electrostatic interactions in a rather generic way by the variable Γ. A main assumption is that the local conformational properties of a polymer change only weakly with changes in the charge distribution, but the global polymer conformations undergo major changes. Then, our results, especially the scaling laws, can easily be connected with other models and experimentally accessible quantities. If our coarse-grained monomers are comprised of *f* “real” monomers, the segment length is *l* = *bf*^*ν*^ in good solvent and the number of monomers of unite charge per polymer is *N*_*m*_ = *N*_*r*_/*f*, where *b* is the segment length of the real polymer and *N*_*r*_ its monomer number. Insertion of these relations into Eq. (11) yields the relationship


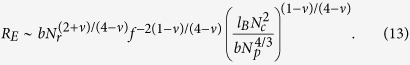


All variables of Eq. (13) can be measured experimentally. The fraction of charged monomer *f*^−1^ is determined by the total number of monomers, the total charge of a nanogel, and the degree of ionization that can be controlled by, e.g., the pH of the solvent.

Our scaling relation is valid only when *N*_*m*_ is larger than about 5. For small *N*_*m*_, inter-blob interactions dominate the swelling behavior. As a consequence, the application of our approach in the limit of weak interaction strengths requires long polymers. Since the polymers in nano- and microgels are often rather short, our approach will typically apply for interactions strengths 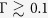, i.e., in the screened regime. Nevertheless, the small-interaction-strength regime is of fundamental theoretical interest and will be useful for novel nano- and microgles comprised long polymers.

In the comparison between scaling theory and simulations, we assumed a scaling exponent *ν* which is independent of the strength of the electrostatic interactions and is approximately equal to the good solvent value *ν* ≈ 0.6. This certainly applies for not to strongly stretched polymers in a gel particle. Indeed, as shown in Fig. 1(c), our polymers are definitely far from being fully stretched. The polymers in the simulations of counterion-free nanogels are far more stretched. Hence, we assume that the applied scaling assumption applies approximately.

Although we focused on salt-free system, our results can safely be extended to systems at moderate salt concentrations. For micrometer size gels, theoretical studies predict that the gel size is nearly constant until the salt concentration *c*_*s*_ becomes comparable to the network charge density *ρ*^40^. Only when *c*_*s*_ surpasses a certain limit, a gel particle strongly shrinks. This is confirmed by experiments^41^,^42^,^43^ and numerical works^44^. Even for nanometer size gels, numerical studies^23^ report that the gel radius exhibits only minor changes and decreases with increasing monovalent salt concentration by only 10% compared to the salt-free radius at *c*_*s*_ ≈ 0.5*ρ*. Additionally, sufficiently large counterion leakage is observed at *c*_*s*_ ≈ 0.1*ρ*^21^. Hence, our scaling considerations should apply up to salt concentrations of approximately 10% of the network charge in nanogels.

We have considered a model nanogel based on an ideal diamond-lattice structure of the crosslinks. Such a regular structure is typically not obtained in the synthesis of gel particles. Although we do not expect this differences to severely influence our qualitative findings, a more realistic description is desirable for a quantitative comparison with experimental results.

It is expected that the interior of a macrogel is neutral even at small Coulomb interaction strengths, in contrast to the considered finite-size nanogel. It remains to be clarified, beyond what gel size the interior of a finite-size particle exhibits macrogel properties. Theoretical considerations indicate that such a crossover occurs at gel radii on the order of 200 nm^19^.

In conclusion, our large-scale molecular dynamics simulations and scaling considerations provide novel insight into the swelling behavior of ionic nanogels. Most importantly, in the *unscreened* regime 4 × 10^−4^ < Γ^*^ < 5 × 10^−2^, Coulomb interactions lead to swelling of the network and its polymers. Here, counterions play a minor role. In the *screened* regime 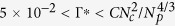, counterions screen the Coulomb interactions and the polymer conformations depend only very weakly on charge interactions. This is quantitatively explained by screening of polyelectrolyte interactions by counterions. The dependence of 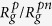 on Γ^*^ suggests that we can control the sensitivity of nanogel swelling to external stimuli through the number of crosslinks *N*_*c*_ and polymers *N*_*p*_. In the *unscreened* regime, nanogels can exhibit major volume changes in response to external stimuli. In contrast, they hardly swell in the *screened* regime even if Γ changes significantly as long as Γ < 1. Thereby, the width of this plateau-like regime broadens with increasing gel particle size, i.e., *N*_*c*_ and *N*_*p*_

## Methods: Model

An individual polyelectrolyte is modeled as a linear chain of *N*_*m*_ monomers of mass *M*, which are connected by the harmonic potential


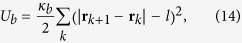


where *l* is the finite bond length^29^. Here, **r**_*k*_ denotes the position of monomer *k* and *κ*_*b*_ is the strength of the bond potential. Excluded-volume interactions are captured by a purely repulsive, truncated, and shifted Lennard-Jones potential^29^. The electrostatic interactions are described by the Coulomb potential


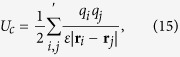


where *q*_*i*_ (*i* ∈ {*m*, *c*}) denotes the electrostatic charge, and the prime indicates *i* ≠ *j*.

A cubic simulation box with periodic boundary conditions is considered. The long-range Coulomb interactions are included by the P2NFFT-algorithm^45^. In the absence of counterions, open boundary conditions are applied. The dynamics of the monomers and counterions are governed by Newton’s equations of motion, which are solved by the velocity-Verlet algorithm^46^. In order to perform isothermal simulations, we couple monomers and counterions with the Brownian multiparticle collision dynamics method (B-MPC)^29^,^30^. B-MPC is a non-hydrodynamic, stochastic, and efficient local thermalization procedure, which provides Maxwell-Boltzmann distributed velocities, and is based on the multiparticle collision dynamics simulation approach for fluids^29^,^47^.

We employ *l* as unit of length, *k*_B_*T* as unit of energy, and 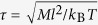 as unit of time. The Lennard-Jones parameters are *σ* = 0.8 *l* and *ε* = *k*_B_*T*. For the spring constant of the bond force, we apply the value *κ*_*b*_ = 10^3^*k*_*B*_*T*/*l*^2^, which ensures nearly rigid bonds with relative bond-length fluctuations below 1%.

## Additional Information

**How to cite this article**: Kobayashi, H. and Winkler, R. G. Universal conformational properties of polymers in ionic nanogels. *Sci. Rep.* **6**, 19836; doi: 10.1038/srep19836 (2016).

## References

[b1] Tanaka T. Collapse of gels and the critical endpoint. Phys. Rev. Lett. 40, 820 (1978).

[b2] Ilmain F., Tanaka T. & Kokufuta E. Volume transition in a gel driven by hydrogen bonding. Nature 349, 400 (1991).

[b3] Das M., Zhang H. & Kumacheva E. Microgels: Old materials with new applications. Annu. Rev. Mater. Res. 36, 117 (2006).

[b4] Saunders B. R. *et al.* Microgels: From responsive polymer colloids to biomaterials. Adv. Colloid Interface Sci. 147–148, 251 (2009). 10.1016/j.cis.2008.08.008 18809173

[b5] Delcea M., Möhwald H. & Skirtach A. G. Stimuli-responsive LbL capsules and nanoshells for drug delivery. Adv. Drug Delivery Rev. 63, 730 (2011). 10.1016/j.addr.2011.03.010 21463658

[b6] Tan B. H. & Tam K. C. Review on the dynamics and micro-structure of ph-responsive nano-colloidal systems. Adv. Coll. Interface Sci. 136, 25 (2008). 10.1016/j.cis.2007.07.002 17707760

[b7] Stuart M. A. C. *et al.* Emerging applications of stimuli-responsive polymer materials. Nature Mater. 9, 101 (2010). 20094081 10.1038/nmat2614

[b8] Thorne J. B., Vine G. J. & Snowden M. J. Microgel applications and commercial considerations. Colloid Polym. Sci. 289, 625 (2011).

[b9] Scherzinger C., Holderer O., Richter D. & Richtering W. Polymer dynamics in responsive microgels: influence of cononsolvency and microgel architecture. Phys. Chem. Chem. Phys. 14, 2762 (2012). 22252036 10.1039/c2cp23328b

[b10] Schneider S. & Linse P. Monte carlo simulation of defect-free cross-linked polyelectrolyte gels. J. Phys. Chem. B 32, 8030 (2003).

[b11] Lu Z. Y. & Hentschke R. Computer simulation study on the swelling of a polyelectrolyte gel by a stockmayer solvent. Phys. Rev. E 67, 061807 (2003). 10.1103/PhysRevE.67.061807 16241254

[b12] Yan Q. & de Pablo J. J. Monte carlo simulation of a coarse-grained model of polyelectrolyte networks. Phys. Rev. Lett. 91, 018301 (2003). 12906581 10.1103/PhysRevLett.91.018301

[b13] Mann B. A., Everaers R., Holm C. & Kremer K. Scaling in polyelectrolyte networks. Europhys. Lett. 67, 786 (2004).

[b14] Edgecombe S. & Linse P. Monte carlo simulations of cross-linked polyelectrolyte gels with oppositely charged macroions. Langmuir 22, 3836 (2006). 16584264 10.1021/la053193i

[b15] Mann B. A., Holm C. & Kremer K. Swelling of polyelectrolyte networks. J. Chem. Phys. 122, 154903 (2005). 15945663 10.1063/1.1882275

[b16] Yin D. W., Horkay F., Douglas J. F. & de Pablo J. J. Molecular simulation of the swelling of polyelectrolyte gels by monovalent and divalent counterions. J. Chem. Phys. 129, 154902 (2008). 19045224 10.1063/1.2991179 PMC2671187

[b17] Quesada-Peres M., Maroto-Centeno J. A. & Martin-Molina A. Effect of the counterion valence on the behavior of thermo-sensitive gels and microgels: A monte carlo simulation study. Macromolecules 45, 8872 (2012).

[b18] Košovan P., Richter T. & Holm C. Molecular simulations of hydrogels. Prog. Colloid Polym. Sci. 140, 205 (2013).

[b19] Claudio G. C., Kremer K. & Holm C. J. Comparison of a hydrogel model to the poisson-boltzmann cell model. J. Chem. Phys. 131, 094903 (2009). 19739869 10.1063/1.3207275

[b20] Jha P. K., Zwanikken J. W., Detcheverry F. A., de Pablo J. J. & de la Cruz M. O. Study of volume phase transitions in polymeric nanogels by theoretically informed coarse-grained simulations. Soft Matter 7, 5965 (2011).

[b21] Jha P. K., Zwanikken J. W., de Pablo J. J. & de la Cruz M. O. Electrostatic control of nanoscale phase behavior of polyelectrolyte networks. Curent Opinion in Solid State and Materials Science 15, 271 (2011).

[b22] Kramarenko E. Y., Khokhlov A. R. & Yoshikawa K. Collapse of polyelectrolyte macromolecules revisited. Macromolecules 30, 3383–3388 (1997).

[b23] Quesada-Pérez M., Ahualli S. & Martn-Molina A. Temperature-sensitive nanogels in the presence of salt: Explicit coarse-grained simulations. The Journal of chemical physics 141, 124903 (2014). 25273470 10.1063/1.4895960

[b24] Denton A. R. Counterion penetration and effective electrostatic interactions in solutions of polyelectrolyte stars and microgels. Phys. Rev. E 67, 011804 (2003). 10.1103/PhysRevE.67.011804 12636524

[b25] Borisov O. & Vilgis T. Polyelectrolyte manifolds. Eur. Phys. Lett. 35, 327 (1996).

[b26] Borisov O. & Daoud M. Scaling theory of branched polyelectrolytes. Macromolecules 34, 8286–8293 (2001).

[b27] Klein Wolterink J., Van Male J., Daoud M. & Borisov O. Starburst polyelectrolytes: Scaling and self-consistent-field theory. Macromolecules 36, 6624–6631 (2003).

[b28] Roger M., Guenoun P., Muller F., Belloni L. & Delsanti M. Monte carlo simulations of star-branched polyelectrolyte micelles. Eur. Phys. J. E 9, 313 (2002). 15010901 10.1140/epje/i2002-10086-0

[b29] Gompper G., Ihle T., Kroll D. M. & Winkler R. G. Multi-particle collision dynamics: A particle-based mesoscale simulation approach to the hydrodynamics of complex fluids. Adv. Polym. Sci. 221, 1 (2009).

[b30] Ripoll M., Winkler R. G. & Gompper G. Hydrodynamic screening of star polymers in shear flow. Eur. Phys. J. E 23, 349 (2007). 17712520 10.1140/epje/i2006-10220-0

[b31] Winkler R. G., Gold M. & Reineker P. Collapse of polyelectrolyte macromolecules by counterion condensation and ion pair formation: a molecular dynamics simulation study. Phys. Rev. Lett. 80, 3731 (1998).

[b32] Kobayashi H. & Winkler R. G. Structure of microgels with debye-hückel interactions. Polymers 6, 1602–1617 (2014).

[b33] Manning G. S. Limiting laws and counterion condensation in polyelectrolyte solutions i. colligative properties. J. Chem. Phys. 51, 924–933 (1969).

[b34] de Gennes P.-G. Scaling Concepts in Polymer Physics (Cornell University, Ithaca, 1979).

[b35] Barrat J.-L. & Joanny J.-F. Theory of polyelectrolyte solutions. Adv. Chem. Phys. 94, 66 (1997).

[b36] Kantor Y. & Kardar M. Excess charge in polyampholytes. EPL 27, 643 (1994).

[b37] Dobrynin A. V., Rubinstein M. & Obukhov S. P. Cascade of transitions of polyelectrolytes in poor solvents. Macromolecules 29, 2974 (1996).

[b38] Limbach H. J., Holm C. & Kremer K. Structure of polyelectrolytes in poor solvent. EPL 60, 566 (2002).

[b39] Doi M. & Edwards S. F. The Theory of Polymer Dynamics (Clarendon Press, Oxford, 1986).

[b40] Barrat J.-L., Joanny J.-F. & Pincus P. On the scattering properties of polyelectrolyte gels. Journal de Physique II 2, 1531–1544 (1992).

[b41] Nisato G., Munch J. & Candau S. Swelling, structure, and elasticity of polyampholyte hydrogels. Langmuir 15, 4236–4244 (1999).

[b42] López-León T., Ortega-Vinuesa J. L., Bastos-González D. & Elassari A. Cationic and anionic poly (n-isopropylacrylamide) based submicron gel particles: Electrokinetic properties and colloidal stability. The Journal of Physical Chemistry B 110, 4629–4636 (2006). 16526694 10.1021/jp0540508

[b43] Capriles-González D., Sierra-Martn B., Fernández-Nieves A. & Fernández-Barbero A. Coupled deswelling of multiresponse microgels. The Journal of Physical Chemistry B 112, 12195–12200 (2008). 18771310 10.1021/jp8003773

[b44] Colla T., Likos C. N. & Levin Y. Equilibrium properties of charged microgels: A poisson-boltzmann-flory approach. The Journal of chemical physics 141, 234902 (2014). 25527958 10.1063/1.4903746

[b45] Pippig M. & Potts D. Particle simulation based on nonequispaced fast Fourier transforms. In Sutmann G., Gibbon P. & Lippert T. (eds) Fast Methods for Long-Range Interactions in Complex Systems, IAS-Series, 131–158 (Forschungszentrum Jülich, Jülich, 2011).

[b46] Allen M. P. & Tildesley D. J. Computer Simulation of Liquids (Clarendon Press, Oxford, 1987).

[b47] Kapral R. Multiparticle collision dynamics: Simulations of complex systems on mesoscale. Adv. Chem. Phys. 140, 89 (2008).

